# Range and climate niche shifts in European and North American breeding birds

**DOI:** 10.1098/rstb.2023.0013

**Published:** 2024-05-27

**Authors:** Damaris Zurell, Katrin Schifferle, Sergi Herrando, Verena Keller, Aleksi Lehikoinen, Thomas Sattler, Levin Wiedenroth

**Affiliations:** ^1^ Ecology and Macroecology Laboratory, Institute for Biochemistry and Biology, University of Potsdam, 14469 Potsdam, Germany; ^2^ European Bird Census Council (EBCC), Prague, CZ-150 00, Czech Republic; ^3^ CREAF, Cerdanyola del Vallès, Barcelona, ES-08193, Spain; ^4^ Catalan Ornithological Institute (ICO), Natural Science Museum of Barcelona, Barcelona, ES-08019, Spain; ^5^ Swiss Ornithological Institute, Seerose 1, 6204 Sempach, Switzerland; ^6^ The Helsinki Laboratory of Ornithology, Finnish Museum of Natural History, University of Helsinki, Helsinki 00014, Finland

**Keywords:** range tracking, niche tracking, transient dynamics, extinction debts, delayed colonization, dispersal limitation

## Abstract

Species respond dynamically to climate change and exhibit time lags. Consequently, species may not occupy their full climatic niche during range shifting. Here, we assessed climate niche tracking during recent range shifts of European and United States (US) birds. Using data from two European bird atlases and from the North American Breeding Bird Survey between the 1980s and 2010s, we analysed range overlap and climate niche overlap based on kernel density estimation. Phylogenetic multiple regression was used to assess the effect of species morphological, ecological and biogeographic traits on range and niche metrics. European birds shifted their ranges north and north-eastwards, US birds westwards. Range unfilling was lower than expected by null models, and niche expansion was more common than niche unfilling. Also, climate niche tracking was generally lower in US birds and poorly explained by species traits. Overall, our results suggest that dispersal limitations were minor in range shifting birds in Europe and the USA while delayed extinctions from unfavourable areas seem more important. Regional differences could be related to differences in land use history and monitoring schemes. Comparative analyses of range and niche shifts provide a useful screening approach for identifying the importance of transient dynamics and time-lagged responses to climate change.

This article is part of the theme issue ‘Ecological novelty and planetary stewardship: biodiversity dynamics in a transforming biosphere’.

## 1. Introduction

Anthropogenic climate change is impacting most ecological processes from genes to ecosystems [1]. Species are responding to climate warming by adapting their phenology, behaviour and physiology, and by shifting their geographical range [2]. Range shifting can lead to massive redistributions of entire species communities with profound effects on ecosystem functioning and human wellbeing, and even feedback loops to the climate system itself [3]. Such range shifts have been observed for different taxa and along latitude, altitude and ocean depth [4–6]. Empirical results suggest that terrestrial species are lagging behind the shifting climate more than marine species [7]. The pace and magnitude of species range shifts is determined by extrinsic factors such as velocity of climate change, and by a number of ecological processes such as dispersal, demography, species interactions and evolution that mediate the species ability to track shifts in climatically suitable areas [8,9]. Understanding such transient and time-lagged dynamics is important for anticipating and when possible attenuating the effects of ongoing environmental change and predicting future biodiversity dynamics [2,8,10].

**Figure 1.**
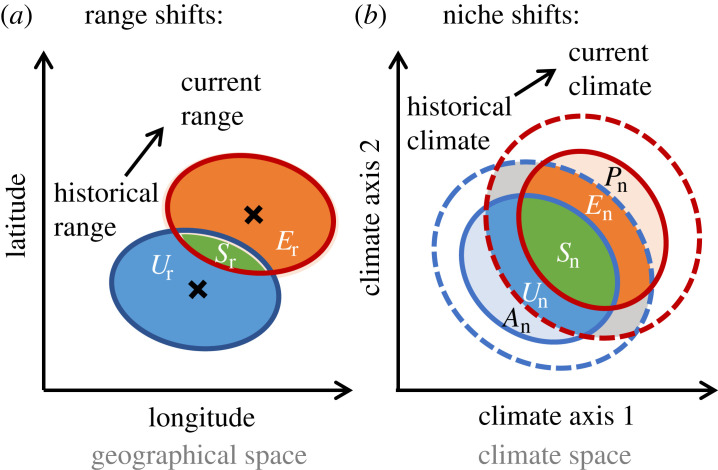
Schematic representation of (*a*) geographical range shift and (*b*) climatic niche shift analyses. Solid lines indicate the historically (blue) and currently (red) occupied range and climate niche, respectively. Dashed lines in the right panel indicate historically and currently available climate. Range and niche dynamics between the two time periods are represented with bold letters. *U*_r_/*U*_n_, range/niche unfilling; *S*_r_/*S*_n_, range/niche stability; *E*_r_/*E*_n_, range/niche expansion. (*b*) In climate space, non-analogue climate conditions in the two time periods could lead to: *A*_n_, niche abandonment; *P*_n_, niche pioneering. Panel (*b*) is adapted from [16,17].

Niche and range dynamics are tightly interlinked. The fundamental niche of a species describes the environmental conditions under which a species can prevail indefinitely. The realized niche of a species may be smaller (or larger) than the fundamental niche owing to demographic and community processes [11–13]. When species are shifting their geographical range in response to climate change, transient population and community dynamics can result in variation of the realized niche over time ([14,15]; figure 1). Niche stability during times of range expansion (colonization of previously unoccupied areas) or range unfilling (retreat from previously occupied areas) would indicate climate tracking. By contrast, unfilling of the realized niche could be indicative of limited dispersal ability and delayed colonization, and expansion of the realized niche could occur owing to slow life-history and extinction debts (i.e. delayed extinction), owing to competitive release or because of adaptation [16]. Quantifying observed range shifts and niche shifts can thus provide a useful screening approach for identifying potential transient dynamics and deriving hypotheses about underlying mechanisms.

The range shifting ability and with it any transient niche shifts are likely to vary among species. Here, we concentrate on macroecological ranges and macroclimatic niches. Range and climatic niche shifts could be affected by biogeographic traits such as latitudinal range centre and range size, and by ecological factors such as body mass, trophic level and habitat preference [18,19]. For instance, we expect that species of higher latitudes show stronger range shifts as the climate is changing faster in higher latitudes and show stronger niche shifts as the higher climate velocities will outpace the range shifting potential [20]. Further, species with larger range size should show higher range and niche stability as the climatic changes experienced by wide-ranging species are small compared with the climatic variation found throughout their range. Similarly, we expect species with wider climatic niches to show stronger niche stability over time. Differences may also emerge from migratory behaviour as sedentary and partially or fully migrant species may use different cues for selecting breeding habitats and thus for shifting their breeding ranges. Body mass and hand wing index (a measure of flight efficiency) are good proxies of movement and dispersal capacity in flying organisms [21–23], and we thus hypothesize that larger species and species with a higher flight efficiency have higher range shifting and niche tracking potential. Furthermore, we assume that a more generalist carnivorous or omnivorous diet may allow species to shift their range and track their climatic niche more easily as they are less dependent on local resources and their prey may also track climatically suitable areas faster than plant species, although we do acknowledge that some prey species may be highly specialized for specific plants which would limit range shifting. Finally, species with different habitat preferences may show distinct range shifting and niche tracking potential. Specifically, we expect variation in range and niche tracking between species that prefer open versus forested habitat although it is difficult to predict which species would exhibit higher or lower range and niche tracking. On the one hand, we could expect that species preferring forest habitats show lower range shifts and higher macroclimatic niche unfilling and niche expansion as the forest habitats may buffer them from climate change and lead to low microclimatic changes. On the other hand, loss of open habitats may be strong owing to increasing farmland intensification and land abandonment resulting in vegetation encroachment, which could both also hamper range shifting [24].

In this study, we aim to quantify and compare the macroecological range dynamics and macroclimatic niche dynamics of breeding birds in Europe and the USA across three decades of climate change from the 1980s until the 2010s. Previous studies suggested that birds of both regions show lagged range shifts [25–27]. We analyse range shifts and climate niche tracking based on kernel density estimation and null model analyses [28]. Finally, we use phylogenetic multiple regression to test the effect of species' biogeography (breeding latitude, range size, climate niche breadth), morphology (body mass, hand wing index as proxy for flight efficiency) and ecology (trophic level, habitat preference, migratory behaviour) on observed range and niche metrics in the two study regions.

## 2. Methods

### (a) Species occurrence data

Occurrence data for European breeding birds were extracted from two European breeding bird atlases (EBBA) with occurrence records from the 1980s (henceforth EBBA1) [29] and 2010s (henceforth EBBA2) [30]. All data were projected to a Lambert azimuthal equal-area (LAEA) projection (ETRS89-extended/LAEA Europe) with 50 km spatial resolution. Species were recorded to be present in a cell if at least one possible breeding attempt was recorded throughout the sampling period. EBBA1 data were mainly collected in 1985–1988, EBBA2 data in 2013–2017. Following the data processing procedure of [26], we completely excluded data from Russia, Kazakhstan, Georgia, Armenia and Azerbaijan, for which the coverage was low and available information was mainly qualitative in EBBA1 [30]. Within the study area, we also excluded cells with low or uneven sampling efforts across the two atlas periods [30].

Occurrence data for United States (US) breeding birds were extracted from the North American Breeding Bird Survey (BBS; www.pwrc.usgs.gov, [31]). The BBS monitoring programme started in the conterminous USA in the 1960s and records bird counts every year during peak breeding season along more than 3000 routes, although not every route is sampled each year. Each route is *ca* 40 km long with 50 equal-spaced stops, at which observers conduct a 3 min count and record every bird species observed or heard within a 400 m radius. Data were downloaded using the R package ‘bbsAssistant’ [32]. For data processing, we followed the protocol by Sofaer *et al*. [33] and restricted the analyses to the conterminous USA where most routes are found. Although occurrence data have been available annually since the 1960s, we picked the temporal range for which annual climatic data were also available and which covers similar decades as the European data. Specifically, we collated data for 3-year time periods in the 1980s (1981–1983) and 2010s (2016–2018). Finally, we included only those routes that had been sampled in all years of both 3-year periods in the 1980s and 2010s, resulting in 521 routes. In accordance with Sofaer *et al*. [33], we chose these 3-year periods as collating data from several years minimizes effects of imperfect detection, while at the same time ensuring that a large number of routes has been surveyed in all 3 years. We only used records at the species level and only included routes of the conterminous USA that meet the BBS methodological criteria (appropriate season, appropriate time of day, appropriate weather conditions, etc.) and were along roadsides (as opposed to waterways). We considered a species present on the route if it was recorded at least once during the 3-year period.

### (b) Climate data extraction

Bioclimatic data were based on the CHELSA climatologies [34]. We downloaded monthly precipitation as well as monthly maximum, minimum and mean temperature for each year of our study periods and the preceding year to account for lag effects of weather on species occupancy [33]. We then calculated the 19 bioclimatic variables [35] using the R package ‘dismo’ [36] and averaged the bioclimatic variables for the study periods (EBBA: 1984–1988 and 2012–2017; BBS: 1980–1983 and 2015–2018).

The climate data were matched to the species data in different ways. For EBBA analyses, CHELSA data were projected using LAEA projection at a resolution of 50 km to match the EBBA grid. For BBS analyses, CHELSA data were projected using Albers equal-area projection (NAD 1983 Albers contiguous USA) at a resolution of 1 km (the original resolution of CHELSA data). Analogous to the procedure by Sofaer *et al*. [33], we extracted climate data from a buffer with a 21 km radius around each route centroid and then averaged the bioclimatic variables within this buffer. Last, for quantifying the global climatic niches of all bird species, CHELSA data were projected to a 50 km global equal-area projection (interrupted Goode homolosine).

### (c) Species filtering and climatic niche coverage

The general workflow is summarized in the electronic supplementary material, figure S1. We only considered the breeding ranges of species, meaning that non-breeding ranges of migratory birds were ignored in our analyses. Prior to quantifying range and niche shifts, we filtered the species according to the following criteria. First, we excluded the pelagic specialists that spend large amounts of time at sea and strongly rely on available shoreline habitat for their breeding colonies. Information about which species forage predominantly pelagic was taken from the Elton Traits 1.0 database [37]. Second, we included only species that were not too rare (e.g. geographical isolates such as island endemics) nor too widespread. For this, species needed to have at least 20 occurrences in each time period to allow statistically sound estimation of the climatic niche [38], and they should not occur in more than 90% of the cells or routes in any of the two time periods. Third, we excluded non-native species. In four cases, species were native to Europe but were introduced in Great Britain or Madeira; we kept those species in the further analyses.

Last, we only included species that had at least 50% of their global climatic (breeding) niche represented in Europe or the USA, respectively. This ensured that the climatic niche shift estimates were representative of the species climatic niches. Previous studies have used the proportion of range covered as criterion [15] but we found this criterion inappropriate for many European birds whose ranges stretch far into Asia. We quantified global climatic niches based on BirdLife range maps [39] (only considering extant, native breeding ranges), which were rasterized at a 50 km spatial resolution using a global equal-area projection (interrupted Goode homolosine). For one species (*Cistothorus platensis*) the BirdLife range map did not overlap with the conterminous USA although the species was recorded in the BBS. This species was excluded from further analysis. Niche coverage (the proportion of global climatic niche represented in the study region; electronic supplementary material, figure S1) was quantified by comparing the regional climatic niche within the European and US study regions, respectively, against the global climatic niche. Niche coverage was quantified for the recent time periods, meaning that niche coverage of European birds and US birds was quantified using climate data for 2012–2017 and 2015–2018, respectively. As the spatial resolution of the BBS data (circular buffers within 21 km radius around the route centroids) did not match the rasterized Birdlife range maps (50 km), the US regional climatic niche was quantified using the Birdlife range maps clipped to the conterminous USA. Regional climatic niches of European birds were determined based on EBBA2 data.

We quantified the niche coverage using the ordination approach of Broennimann *et al*. [28] within the ‘ecospat’ R package [40] (electronic supplementary material, figure S1). First, we summarized the global climate space by the first two axes of a principal component analysis (PCA) over all 19 bioclimatic variables. For European breeding birds, the first two PCA axes explained 58–86% (mean 70%) of the climatic variation in the global ranges and for North American birds 58–83% (mean 71%). Second, for each species we estimated the density of regional occurrences in climate space and the density of global occurrences in climate space using kernel density estimation. Species densities were corrected for availability of climate space by dividing the density of presences by the density of the environment (the total global climate space) [28]. Third, we overlayed the regional occurrence densities of Europe and USA, respectively, with the global occurrence densities in climate space and quantified the proportion of regional occurrence densities overlapping with the global occurrence densities, which served as our measure of climatic niche coverage (electronic supplementary material, figures S1 and S2).

After all these filtering steps, 114 European and 195 North American breeding bird species were left for analyses of range and niche shifts (electronic supplementary material, figures S3 and S4).

### (d) Range and niche overlap analyses

First, we quantified for each species the Euclidean distance between the range centroids of both study periods and the direction of range shifts in degrees. Then, range and niche overlap analyses were based on procedures from [28,41] using the ‘ecospat’ R package [40]. These use kernel density estimators to quantify the density of historical and current occurrences in two-dimensional space. The ranges were quantified in the two-dimensional geographical space while the niches were quantified along the first two axes of a PCA over all 19 bioclimatic variables (as was done for the global climate niche quantification). For both range and niche estimation, we restricted the background environment to those cells within a 500 km buffer around the presence cells for each species, to account for a species' historical ability to reach those places by dispersal. Species densities were corrected for availability of environment by dividing the density of presences by the density of the background environment [28]. We then calculated range overlap and niche overlap between the two time periods using Schoener's *D* metric [42]. This metric ranges between 0 (no overlap) and 1 (complete overlap).

We also calculated the range dynamic metrics: range unfilling (*U*_r_), range stability (*S*_r_) and range expansion (*E*_r_) (figure 1*a*) as the proportion of occurrence densities in geographical space that were only occupied in the historical time period, occupied in both time periods, and only occupied in the current time period, respectively. The niche dynamic metrics: niche unfilling (*U*_n_), niche stability (*S*_n_) and niche expansion (*E*_n_) in climate space were calculated analogously. All range dynamic metrics and niche dynamic metrics were standardized to sum up to 1 (*U*_r_ + *S*_r_ + *E*_r_ = 1 and *U*_n_ + *S*_n_ + *E*_n_ = 1) for easier interpretation. Additionally, as sensitivity analysis, we also quantified the niche dynamic metrics niche abandonment (*A*_n_) as the proportion of occurrence densities that occupied climatic conditions in the historical time period that had no climate analogue in the current time period, and niche pioneering (*P*_n_) as the proportion of occurrence densities that occupied climatic conditions in the current time period that had no climate analogue in the historical time period (figure 1*b*). However, as niche abandonment and niche pioneering tended towards zero, we only present the results within the analogue climate space.

Finally, we used similarity tests to assess significant range lagging and range switching, and significant niche tracking and niche switching. Significant range lagging means that the historical range is preserved into the current time period, while significant range switching means that historical and current range are more dissimilar than expected by chance. Analogously, significant niche tracking means that the historical niche is conserved while significant niche switching means that the historical and current realized niche are more dissimilar than expected by chance. Similarity tests use a null model approach in which the occurrence densities of one time period are shifted randomly in geographical or climate (PCA) space, respectively. Then the range and niche dynamic metrics are recalculated comparing the simulated densities against the observed densities of the other time period [28]. Here, the historical range and niche were kept constant while the recent ones were randomly shifted. We ran similarity tests with 1000 iterations in geographical space and 1000 iterations in climate space to test whether range and niche metrics were higher or lower than expected by chance. We tested the metrics niche/range overlap, stability, expansion and unfilling (cf. figure 1) in the similarity tests. An interpretation guide is summarized in the electronic supplementary material, table S1.

### (e) Trait analyses

We used phylogenetic regression to test our hypotheses and assess the covariation of range and niche metrics with morphological, ecological and biogeographic traits. Trait information was extracted from the AVONET database [43]: body mass, hand wing index, trophic level (ordinal scale: 1 = herbivore, 2 = omnivore, 3 = carnivore and scavenger), migratory behaviour (ordinal scale from 1 = sedentary to 3 = migratory), habitat openness (termed habitat density in AVONET; ordinal scale from dense habitats = 1 to open habitats = 3), range size and latitudinal range centroid. Additionally, we calculated global climatic niche breadth following [44,45]. For this, we used the rasterized Birdlife range maps (50 km), estimated the density of occurrences in two-dimensional climate (PCA) space and corrected for climate availability, taking the entire globe as background environment to ensure that niche size was comparable across species. Climatic niche breadth was then defined as the Shannon index calculated from the occurrence densities in climate space (using the ‘vegan’ R package [46]). The Shannon index considers both the size of the occupied climate space and the evenness in occupancy. Phylogenetic information was extracted from Jetz *et al*. [47]. For some species that were more recently recognized as sister species, no separate phylogenetic information was available and they were thus excluded from trait analyses. Overall, 111 European and 188 North American breeding bird species were included in the trait analyses.

We estimated separate phylogenetic regression models for each region (Europe, USA), with Akaike information criterion (AIC)-based variable selection, the different niche and range dynamic metrics (unfilling, stability, expansion) as response variable and the traits as independent variables, while controlling for phylogenetic relatedness. All models were estimated using the ‘phylolm’ R package [48]. As all niche and range metrics varied between 0 and 1, we logit-transformed them prior to modelling. All trait variables were centred and standardized. Phylogenetic signal was estimated using Pagel's *lambda* [49] where 0 implies no phylogenetic signal and 1 implies high phylogenetic signal evolved under Brownian motion. Variable importance was quantified by randomly permutating each predictor variable and assessing the mean drop in explained variance over *n* = 99 iterations [50].

## 3. Results

In Europe, climatic changes over the study period varied from south or southwest to north or northeast. Specifically, the climate changed towards a warmer and drier climate in the western Mediterranean and central Europe and towards a wetter climate in the eastern Mediterranean and Scandinavia, and towards less seasonality in northern Europe (electronic supplementary material, figure S5). In the USA, we found a strong east-west gradient of climate change, with a change towards warmer and wetter climate in the east, and towards drier and less seasonal climate in the west (electronic supplementary material, figure S6). These climatic changes coincided with distinct range shifts for species in both study regions. Most European birds shifted their ranges in a northern to north-eastern direction, on average by 70 km (ranging 4–466 km; electronic supplementary material, figure S7). By contrast, most US birds shifted their ranges in a western direction, on average by 125 km (ranging 9–760 km; electronic supplementary material, figure S8).

**Figure 2.**
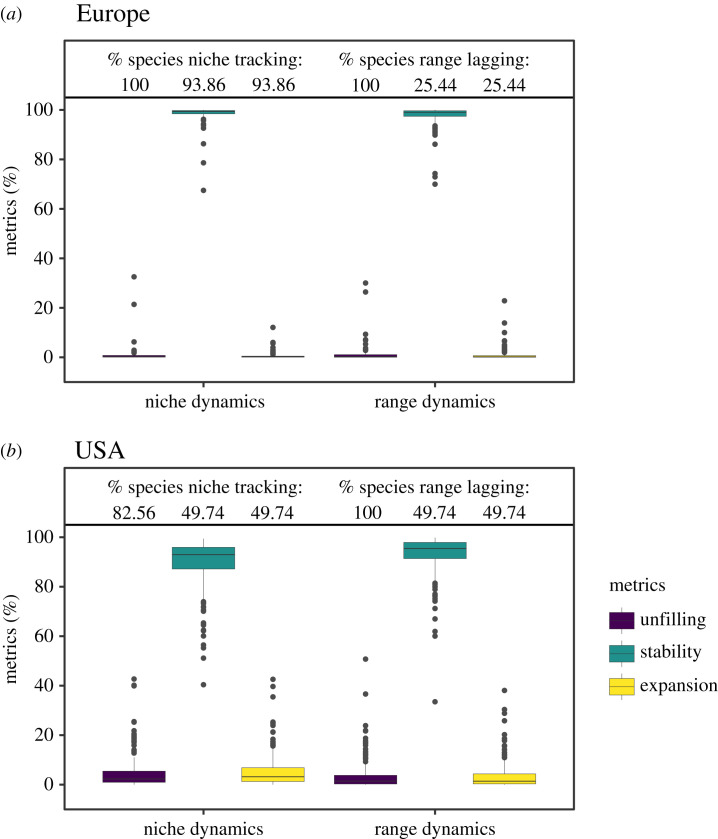
Niche and range dynamics for (*a*) European (*n* = 114) and (*b*) North American breeding birds (*n* = 195). The different niche and range metrics are explained in figure 1. Significance was tested by similarity tests (*n* = 1000 replicates).

Range overlap and niche overlap estimated by Schoener's *D* between the historical and recent study periods were high in both regions, although significantly higher for European birds than for US birds (electronic supplementary material, figures S9–S10). The range dynamic metrics indicated that the species geographical ranges in Europe and the USA have remained largely stable with relatively little range unfilling and range expansion (figure 2). Yet, similarity tests revealed that only *ca* 25% of European birds and *ca* 50% of US birds showed significantly higher range stability than expected by chance (compared to random reshuffling of the ranges within the available geographical space). This indicates that significant range lagging was comparably low (figure 2). Thereby, more species tended to expand their range than contract their range, indicated by 100% of species showing significant range lagging in terms of range unfilling compared to 25–50% range lags in terms of range expansion. We found no significant range switching. Also, we found no correlation between range unfilling and range expansion, meaning that extirpation from previously occupied places and colonization of newly suitable places were largely independent (electronic supplementary material, figure S11).

Differences in niche dynamic metrics were more pronounced between the study regions. Most European species showed large niche stability and almost no niche unfilling and niche expansion, and the similarity tests indicated niche conservatism for at least 94% of the species (figure 2). By contrast, niche dynamic metrics were more variable in the USA, although niche stability was still considerably higher than niche unfilling and niche expansion. Also, the similarity tests indicated lower niche tracking in the USA with only 50% of the species showing significantly higher niche stability and significantly lower niche expansion than expected by chance, while 83% of species showed significantly lower niche unfilling (figure 2). Results were similar when considering a slightly shorter time difference between the historical and recent time period for US breeding birds that is more comparable to the European data (electronic supplementary material, figure S12). We found consistent correlation patterns between range dynamic and niche dynamic metrics for the two study regions (electronic supplementary material, figures S13–S14). Notably, there was no significant linear correlation between range expansion and niche unfilling, nor between range unfilling and niche expansion. Nevertheless, niche unfilling only occurred when range expansion was low. Range unfilling and niche expansion showed a weak positive correlation for US breeding birds but not for European birds (electronic supplementary material, figures S13–S14).

**Figure 3.**
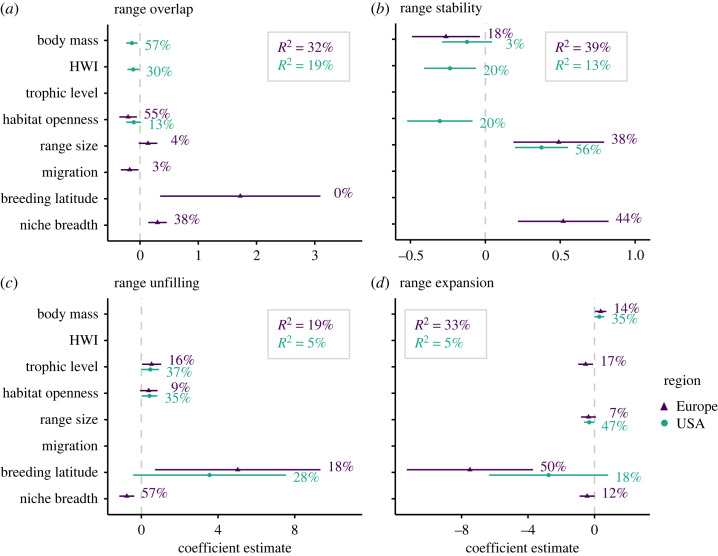
Trait effects on range metrics. To explain how the different range metrics relate to species morphological, ecological and biogeographic traits, we ran phylogenetic regression models using AIC-based variable selection for the two different regions (Europe, conterminous USA). We only considered linear effects of the different traits. Error bars represent the coefficient means and their 95% confidence intervals. Percentages indicate the variable importance, estimated by random permutation of each variable (*n* = 99 iterations). *R*^2^ refers to the overall explained variance of the models. HWI, hand wing index.

In the USA, the selected traits explained 5–19% of the variance in range metrics, while in Europe traits explained 19–39% of the variance (figure 3). Also, variable importance differed markedly between the two regions. Range overlap in US breeding birds was best explained by a negative effect of body mass, hand wing index and habitat openness. By contrast, range overlap in European breeding birds was best explained by a negative effect of habitat openness and a positive effect of climate niche breadth. Range stability in the USA significantly increased with range size and significantly decreased with habitat openness and hand wing index. In comparison, range stability in Europe significantly increased with niche breadth and range size, and significantly decreased with body mass. Range unfilling increased with breeding latitude, habitat openness and for higher trophic levels in both regions. Additionally, in Europe, range unfilling significantly decreased with climate niche breadth. Range expansion in Europe and in the USA decreased with breeding latitude and range size and increased with body mass. In Europe, we also found considerable negative effects of trophic level and climatic niche breadth on range expansion.

**Figure 4.**
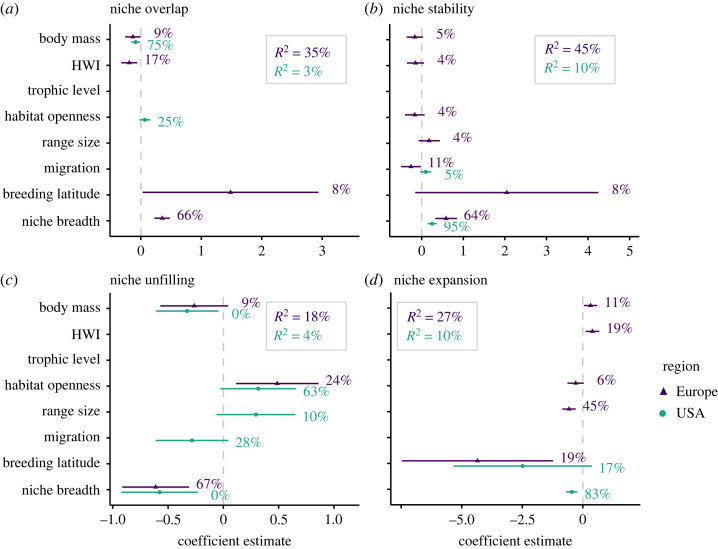
Trait effects on niche metrics. To explain how the different niche metrics relate to species morphological, ecological and biogeographic traits, we ran phylogenetic regression models using AIC-based variable selection for the two different regions (Europe, conterminous USA). We only considered linear effects of the different traits. Error bars represent the coefficient means and their 95% confidence intervals. Percentages indicate the variable importance, estimated by random permutation of each variable (*n* = 99 iterations). *R*^2^ refers to the overall explained variance of the models. HWI, hand wing index.

Traits also explained a large proportion of variation in niche overlap and niche dynamic metrics of European birds, with a range of 18–45%, while overall explained variance in niche metrics of US breeding birds was low, with a range of 3–10% (figure 4). Niche overlap in the USA was largely explained by a negative effect of body mass and a positive effect of habitat openness. By contrast, niche overlap in Europe significantly increased with climate niche breadth and breeding latitude, and decreased with body mass and hand wing index. Variation in niche stability was best explained by a positive effect of niche breadth for both European and US birds. The effect of migration differed between regions with a positive effect of migration ability in the USA but a negative one in Europe. While a negative effect of niche breadth best explained niche unfilling in Europe, habitat openness, range size and migration ability were far more important in the USA. Niche expansion in the USA was negatively affected by climate niche breadth and by breeding latitude. In Europe, niche expansion decreased with range size, breeding latitude and habitat openness, and increased with body mass and hand wing index.

## 4. Discussion

Our comparison of range and niche shifts in European and US breeding birds over three decades of climate change revealed important similarities and differences in range and niche dynamics in these regions. The direction of climate change and range shifts differed strongly between regions, potentially indicating different magnitudes of transient phenomena. Still, some common patterns emerged. Notably, in both regions we observed significantly lower range unfilling than expected by chance, indicating that decolonization was slow while range expansions were more common. Also, niche expansions, which can be indicative of extinction debts (i.e. delayed extinction), competitive release or adaptation, were more common than niche unfilling that is indicative of dispersal limitations. This could indicate that European and North American birds were not dispersal limited over the study period. The climatic niches remained more stable over time in Europe compared to the USA. This could suggest that transient dynamics are more pronounced in the USA but it could also reflect differences in underlying data. Traits related to the morphology, ecology and biogeography of species explained much more of the variation in range and niche metrics in European birds than in US birds and we found partially contrasting trait effects in these two regions. This suggests important differences in the mechanisms underlying range and niche dynamics in Europe and the USA [51], related for example to different orientation of main mountain chains (Europe east-west, US north-south) [52] and to different land use constraints.

Range shifts were observed in both regions although US breeding birds showed larger range shifts on average. The European birds considered here shifted their ranges mostly in a  northern to north-eastern direction, which is in line with recent findings from all European birds [26] and in line with the geography of European main mountain chains. By contrast, US birds shifted mostly in a western direction. This phenomenon has also been reported earlier in 21 passerine species [25] and our results show that this finding is generalizable to the larger bird species pool of conterminous USA and probably related to continent specific orography. However, we note that these results are not generalizable beyond the considered study regions because of the geographical configuration of these and neighbouring land masses. Many breeding birds within the USA have ranges that stretch farther north into Canada where more northward range shifts are possible. Likewise, many breeding birds within Europe have ranges that stretch farther east into Asia where more eastward range shifts are possible. In the future, analyses on the full geographical ranges of these birds would be desirable.

In both regions, large proportions of the species geographical ranges remained relatively stable while comparably small areas became newly occupied through range expansion or unoccupied through range unfilling. Interestingly, most species showed significantly lower range unfilling than expected by chance while only 25–50% of species showed significantly lower range expansion than expected by chance, indicating that unoccupied places are more likely to become occupied than occupied places are to becoming unoccupied. This was especially pronounced in Europe. Our study did not explicitly distinguish leading and trailing range edges. In the future, it would be desirable to ascertain whether trailing range edges were more inert than expanding range fronts [53–55]. Lower range expansion than expected could indicate a role of extinction debts [56]. Extinction debts and delayed colonization in US birds have also been found in [57]. Our results corroborate these findings and suggest that time delays in extinctions were higher than in colonization, and that European and US birds do not seem to be dispersal limited (but see [52]). Importantly, niche expansions and low range unfilling could indicate extinction debts, but also competitive release, tracking of non-climatic factors, or adaptation, which cannot be disentangled with the simplified screening method used here. An earlier study on Californian birds found high rates of niche stability and niche tracking [58]. While our results corroborate the finding of high niche stability, they also revealed that niche conservatism was much less pronounced in US birds. This could indicate stronger transient dynamics compared to European birds but could also result from differences in the data observation process with lower detection probability in the USA that could lead to lower estimates of niche stability.

We tested several hypotheses related to the effects of morphological, ecological and biogeographic traits on range and niche dynamics using phylogenetic regression. In terms of biogeographic traits, we found limited support for our hypothesis that species of higher latitudes show stronger range shifts. On the contrary, range expansion was significantly lower for birds breeding in higher latitudes and breeding latitude was even the most important predictor of range expansion in Europe. This effect could be a result of limited geographical space available to range expansion. In Europe, the continental boundaries prohibit further northward range expansion. In the USA, it could indeed be an artefact as the analyses were limited to conterminous USA, while the ranges of the bird species may stretch into Canada and could also further expand there. Range unfilling was significantly higher for species of high latitudes in both regions, meaning that they retreated from previously climatically suitable areas faster. Also, we must refute our hypothesis of stronger niche shifts in high-latitude species. Indeed, niche expansion significantly decreased with breeding latitude. This could indicate that northern-ranging species exhibit lower extinction debts and together with the higher range unfilling in higher latitude species this could suggest more rapid replacement by competitively stronger, warm-adapted species at the trailing range edges. Current empirical evidence for such replacement is not unequivocal [59,60] and hence warrants further investigation. As expected, we found higher range and niche stability for wide-ranging species and for species with wider climatic niches, which is well in line with previous findings [19]. In contrast to other studies [61], we found that niche unfilling in US birds decreased from sedentary to fully migrant birds, indicating higher dispersal limitations in sedentary birds compared to migrants.

Our hypotheses related to the effects of morphological and ecological traits were only partially supported and we found pronounced differences between the two regions. As body mass and hand wing index (flight efficiency) are good proxies of dispersal ability [21,62] we expected to find higher range shifting and higher niche tracking potential for large-bodied species and efficient flyers. In support of this hypothesis, range stability significantly decreased with body mass in both regions, and in the USA it also decreased with hand wing index. Yet, contrary to expectations, niche overlap and niche stability significantly decreased with body mass. Still, niche unfilling of European birds decreased with body mass as expected indicating that large-bodied species exhibit lower dispersal limitations. In Europe, niche expansion significantly increased with body mass and hand wing index while there was no such effect in the USA. Here, the positive effect of body mass on niche expansion could indicate that large-bodied species that often have slow life histories and longer generation times may show higher extinction debts [63]. To disentangle these effects, it would be desirable to have data on dispersal ability to avoid relying on proxies [64].

We expected that a more generalist omnivorous or carnivorous diet may allow stronger range shifting and higher niche tracking but found weak support for this hypothesis. By contrast, we found the opposite in Europe with lower range expansion and thus more inert range edges for carnivores and omnivores compared to herbivores. Still, carnivorous species showed significantly higher range unfilling, meaning they retreated faster from previously occupied ranges. Thus, (temporary) persistence in climatically unsuitable areas and, hence, extinction debts could be more pronounced in herbivores than in omnivores and carnivores, which may be explained by extinction debts of the plant resources [65,66]. Last, we found that species in dense forest habitats showed lower range shifts. Range overlap significantly decreased with preference for more open habitats in both regions. In the USA, the same effect was found for range stability. Also, range unfilling significantly increased with habitat openness in both regions, meaning that species of more open habitats retreated faster from previously occupied ranges. Relatively longer persistence of birds preferring dense forests could relate to a microclimatic buffering effect from macroclimatic change [67]. Also, niche unfilling significantly increased with habitat openness in both regions. This indicates stronger dispersal limitations for open-habitat species, either because of limited dispersal ability or because of competitors blocking the range front. Limited dispersal ability could to some extent also be related to land use practises, e.g. farmland intensification, leading to fragmentation and patchy distribution of natural open habitats [68] but also to population decreases of farmland birds. We deliberately concentrated on climate change in this study but in the future, the joint effects of climate and land use change should be further investigated.

Overall, our study suggests similarities but also important differences in range and niche dynamics of European and US breeding birds. In part, this could be explained by differences in geography (e.g. mountain chains) and climate and thus differences in the trajectories, magnitudes and directions of climate change in these two continents [51]. Also, both regions have very different land use histories [69], which we did not explicitly test here. Our study analysed range and niche dynamic metrics as simple proxies of transient dynamics borrowed from invasion science [16]. This allows deriving hypotheses about underlying mechanisms related, for example, to extinction debts and dispersal limitations, but further research is needed to affirm the hypothesized mechanisms. Previous analyses based on correlative species distribution models indicated that observed range shifts did not align with predicted range shifts expected under the observed climatic changes [26,27]. Our results can neither corroborate nor refute these findings but suggest time-lagged responses and transient dynamics as potential explanation for such misalignment. In the future, mechanistic models that explicitly consider important ecological processes such as dispersal, demography and species interactions [8,13] could help in attributing trends to different abiotic drivers and biotic processes [70,71].

## Data Availability

The occurrence data from BBS and EBBA are available open access through their dedicated repositories. We provide codes for repeating all analyses and any intermediate and final results in the Zenodo Repository: https://doi.org/10.5281/zenodo.8403278 [72]. Supplementary material is available online [73].
